# Parkinson’s disease patients show delayed hemodynamic changes in primary motor cortex in fine motor tasks and decreased resting-state interhemispheric functional connectivity: a functional near-infrared spectroscopy study

**DOI:** 10.1117/1.NPh.11.2.025004

**Published:** 2024-05-29

**Authors:** Edgar Guevara, Francisco Javier Rivas-Ruvalcaba, Eleazar Samuel Kolosovas-Machuca, Miguel Ramírez-Elías, Ramón Díaz de León Zapata, Jose Luis Ramirez-GarciaLuna, Ildefonso Rodríguez-Leyva

**Affiliations:** a CONAHCYT-Universidad Autónoma de San Luis Potosí, San Luis Potosí, Mexico; b Universidad Autónoma de San Luis Potosí, Coordinación para la Innovación y Aplicación de la Ciencia y la Tecnología, San Luis Potosí, Mexico; c Hospital Central “Dr. Ignacio Morones Prieto”, Universidad Autónoma de San Luis Potosí, Faculty of Medicine, Neurology Service, San Luis Potosí, Mexico; d Universidad Autónoma de San Luis Potosí, Faculty of Science, San Luis Potosí, Mexico; e Tecnológico Nacional de México, San Luis Potosí, Mexico; f Hospital Central “Dr. Ignacio Morones Prieto”, Universidad Autónoma de San Luis Potosí, Division of Surgery, Faculty of Medicine, San Luis Potosí, Mexico

**Keywords:** Parkinson’s disease, functional near-infrared spectroscopy, cortical activity, resting state, functional connectivity

## Abstract

**Significance:**

People with Parkinson’s disease (PD) experience changes in fine motor skills, which is viewed as one of the hallmark signs of this disease. Due to its non-invasive nature and portability, functional near-infrared spectroscopy (fNIRS) is a promising tool for assessing changes related to fine motor skills.

**Aim:**

We aim to compare activation patterns in the primary motor cortex using fNIRS, comparing volunteers with PD and sex- and age-matched control participants during a fine motor task and walking. Moreover, inter and intrahemispheric functional connectivity (FC) was investigated during the resting state.

**Approach:**

We used fNIRS to measure the hemodynamic changes in the primary motor cortex elicited by a finger-tapping task in 20 PD patients and 20 controls matched for age, sex, education, and body mass index. In addition, a two-minute walking task was carried out. Resting-state FC was also assessed.

**Results:**

Patients with PD showed delayed hypoactivation in the motor cortex during the fine motor task with the dominant hand and delayed hyperactivation with the non-dominant hand. The findings also revealed significant correlations among various measures of hemodynamic activity in the motor cortex using fNIRS and different cognitive and clinical variables. There were no significant differences between patients with PD and controls during the walking task. However, there were significant differences in interhemispheric connectivity between PD patients and control participants, with a statistically significant decrease in PD patients compared with control participants.

**Conclusions:**

Decreased interhemispheric FC and delayed activity in the primary motor cortex elicited by a fine motor task may one day serve as one of the many potential neuroimaging biomarkers for diagnosing PD.

## 1 Introduction

Parkinson’s disease (PD) is a progressive neurological condition that affects movement. The condition is due to the loss of neurons in the substantia nigra of the brain, which is the area responsible for the production of dopamine. This neurotransmitter is essential for movement. The loss of neurons causes a reduction in dopamine production, leading to the characteristic symptoms of the condition: tremors, rigidity, bradykinesia, and postural instability.^1^ As of today, there is no cure for PD, but treatment that can provide significant relief from symptoms is available, and neuroimaging techniques can be used to monitor the progress of the disease and predict its severity. Therefore, the diagnosis of PD is based on the presence of these symptoms, together with a detailed history of the patient’s condition.^2^ The Unified Parkinson’s Disease Rating Scale (UPDRS) is widely used for diagnosing PD.^3^ The criteria are based on tremor, rigidity, bradykinesia, and postural instability.

The condition has been well studied, and there is a good understanding of the underlying causes. There is, however, a need for new diagnostic techniques that are objective and provide a measure of disease severity. New neuroimaging techniques are needed for the diagnosis and monitoring of PD progression due to the complexity and variability of the disease. Current imaging techniques may not fully capture the early stages or the full spectrum of PD symptoms.^4^ One such technique is functional near-infrared spectroscopy (fNIRS), which is a portable, non-invasive optical neuroimaging technique that measures changes in brain activity by estimating oxygenated and deoxygenated hemoglobin near the brain’s surface, thus enabling the investigation of cortical activity, which can be altered in PD. At the same time, participants move freely.^5^ Unlike traditional imaging methods such as functional magnetic resonance imaging (fMRI), fNIRS allows participants to move and speak relatively easily, making it well suited to exploring the full complement of neuroimaging research questions and serving as a viable alternative to fMRI due to its unique advantages such as portability, resiliency to motion artifacts, and tolerance to metallic implants; these make it more suitable for patients with PD who may have difficulty remaining still or have deep brain devices not compatible with fMRI.^5^^,^^6^ In addition, fNIRS is safe, portable, lightweight, cost-effective, and less sensitive to motion artifacts and environmental noise than other imaging techniques.^7^^,^^8^

fNIRS can potentially study movement disorders such as PD as it can overcome movement restrictions and enable experiments in more natural conditions.^9^ Focusing specifically on fine motor skills, fNIRS has been used to discriminate between different motor responses and levels of motor complexity, making it a relevant tool for monitoring changes related to fine motor skills.^10^ In addition, fNIRS is a promising approach to studying the presumed contribution of dysfunction within the pre-frontal cortex (PFC) to difficulties in PD patients.^11^ Furthermore, fNIRS has been used to assess fine motor tasks in PD patients during stepping tasks^12^ and under deep brain stimulation.^13^ It has been shown that fNIRS can detect changes in cortical activation patterns during fine motor tasks in PD patients. For instance, Bonilauri et al.^14^ found that early-stage PD patients showed higher activation in motor and occipital areas compared with those with moderate PD, who had increased activation in frontal areas, suggesting a shift in brain activation patterns with disease progression. Simpson and Mak^15^ suggested that transcranial direct current stimulation alters motor cortex oxygenation in both PD patients and healthy controls but does not modulate its task-related activity.

When assessing the cognitive status of PD patients, research has shown fine motor skills to be impaired. Furthermore, this impaired cognitive status may exacerbate such difficulties in PD patients.^16^ Using fNIRS to measure cortical blood flow changes related to fine motor skills, researchers can assess relevant motor symptoms in PD patients and determine whether patients have mild cognitive impairment or not.^17^ Overall, fNIRS has the potential to study fine motor tasks in PD patients and to monitor changes related to the development of the disease.

Furthermore, fNIRS can assess the brain’s resting-state functional connectivity (RSFC). RSFC measures the temporal correlation among spatially remote brain regions that exhibit synchronous low-frequency fluctuations in blood oxygenation level-dependent signals.^18^ RSFC has been used to compare differences in functional connectivity (FC) between patients with PD and age-matched controls using fMRI.^19^ Comparing RSFC between these two groups can provide insights into the pathophysiology of PD and help identify potential biomarkers for early diagnosis and treatment.^19^

In the present study, fNIRS was used to examine the hemodynamic response to fine motor skills in the motor cortex of PD patients and control participants. First, correlations between the hemodynamic response function (HRF) parameters and clinical markers were analyzed. Thereafter, motor cortex activity during a walking task was measured, and the RSFC in these patients was assessed.

## 2 Methods

### 2.1 Ethics Statement

The study protocol was designed according to the Declaration of Helsinki and reviewed and approved by the Institutional Review Board (registration number 77-21). The study was conducted at the Neurology Department of the Central Hospital “Dr. Ignacio Morones Prieto” in Mexico. The recruiting period was from October 2021 to October 2022. Before testing, all participants provided signed informed consent.

### 2.2 Participants

Twenty patients were enrolled in our clinic and were diagnosed according to the criteria of the United Kingdom PD Society Brain Bank.^20^ All PD patients were on anti-Parkinson drugs. Daily Levodopa equivalent doses were determined from total daily doses for participants using anti-Parkinson medicines using a conversion formula.^21^ In the present analysis, we included patients with a tremor-dominant (N=11) or akinetic-rigid subtype (N=9).^22^ A neurologist (FJRR) used the Hoehn and Yahr (HY) scale^23^ and the Movement Disorder Society-Sponsored Revision of the Unified Parkinson Disease Rating Scale (MDS-UPDRS, part III) to determine the severity of the disease.^3^ Freezing of gait severity was assessed with the freezing of gait questionnaire (FOGQ).^24^ All evaluations detailed in this study were conducted while the subjects were in their “off” medication state (∼12  h after the last anti-parkinsonian medication dose was taken). The MDS-UPDRS part III scores were additionally validated by an independent rater.

The control group was composed of 20 age- and sex-matched healthy participants without movement disorders. All participants underwent the Spanish version of the Montreal Cognitive Assessment (MoCA) for global cognitive function assessment.^25^ Severe cognitive impairment at baseline was defined as a score <10 on the MoCA examination, and individuals with this score or below were excluded from this study.

### 2.3 fNIRS Data Acquisition

**Fig. 1:**
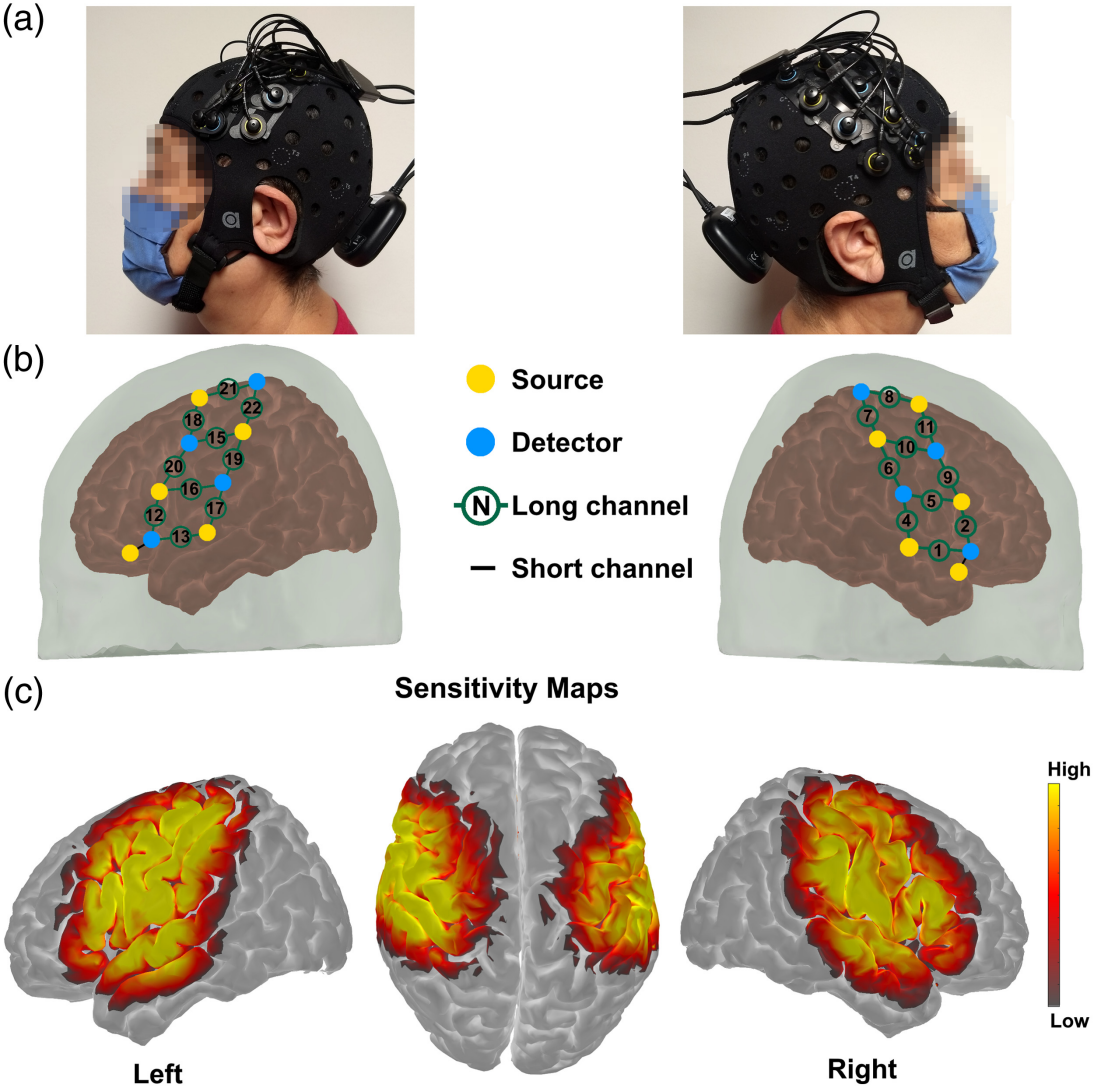
(a) Photographs of optode array placement on one participant’s head. (b) Registered probe geometry: yellow dots indicate the 10 sources, and cyan dots specify the eight detectors. Black numbers in green circles indicate long separation channels, and the black lines denote SSCs. (c) Sensitivity profile displayed in a log10 scale.

The portable fNIRS system Brite MKII (Artinis Medical Systems BV, the Netherlands) was employed for measuring cortical brain activity in the present study. This system employs 10 dual-wavelength light-emitting diodes (LEDs) centered at 757 and 843 nm, with a sampling frequency of 25 Hz, which is sufficiently high enough to avoid aliasing effects of blood pressure, respiratory, and cardiac frequencies. Participants in the study were outfitted with a black neoprene head cap, with sizes varying between 54 and 60 cm, based on their head circumference, as depicted in Fig. 1(a). The cap was placed on the head of the participant, and the fNIRS optodes were securely held throughout data collection. There were eight detectors and 10 near-infrared sources; the optodes were placed 3 cm apart for the 20 long channels and 1.5 cm apart for the two short channels. The sources and detectors were positioned in the cap holders before the cap was worn by the participant and then manually adjusted to achieve an efficient optode-scalp coupling, as determined in real time by the acquisition software (Oxysoft, Artinis Medical Systems BV, the Netherlands). A three-dimensional digitizer (Patriot, Polhemus Inc., Colchester, Vermont, United States) was used to record the position. The 20 long channels [10 per hemisphere, as shown in Fig. 1(b)] were utilized to measure changes in hemoglobin, which serves as a proxy measure of brain activity, across bilateral motor regions of the brain [Fig. 1(c)], and the two short channels were used to minimize the effects of superficial hemodynamics.^26^^,^^27^

### 2.4 Experimental Protocol 1: Fine Motor Task

**Fig. 2:**
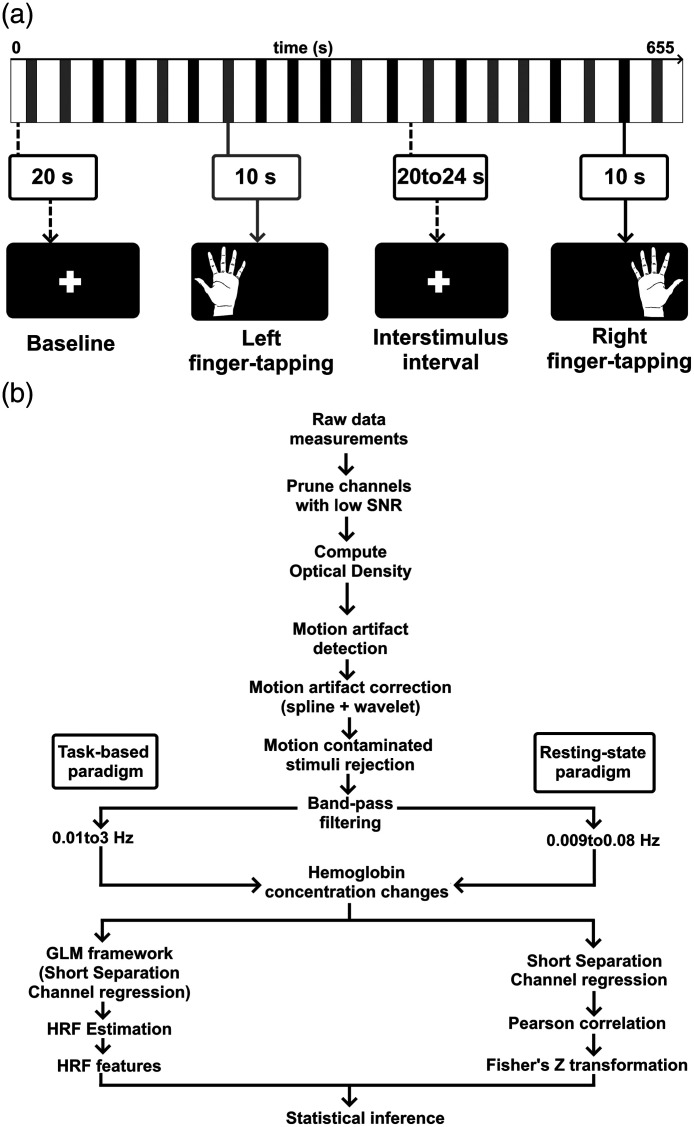
(a) Visualization of the experimental protocol, which includes two types of finger-tapping tasks: left and right. The entire experiment took 11 min. (b) Flow chart of the processing steps used in this study.

A finger-tapping exercise activated the primary motor cortex (M1), and its task-related hemodynamic activity was measured. Next, subjects were told to tap their thumbs consecutively with the other fingers of the same hand (i.e., thumb and index finger tapping, thumb and middle finger tapping, etc.). The action was performed in brief bursts of 10 s, interspersed with rest periods ranging from 20 to 24 s. This variable interstimulus period was intended to prevent task events from becoming phase-locked to physiological hemodynamic oscillations^28^ and to reduce anticipatory effects.^29^ In addition, video cues were used to instruct movement initiation and offset. The motor task lasted around 11 min and was divided into 20 blocks (10 with the left hand and 10 with the right hand, randomly allocated), as depicted in Fig. 2(a).

### 2.5 Experimental Protocol 2: A 2-min Walk

The participants initially conducted a 2-min walking exercise with a baseline standing period of 20 s. The walking response was analyzed as a single trial from −2 to 25 s, but 2 min were allocated to see if the patients presented gait freeze. During the over-ground walking condition, participants walked back and forth along a 6 m straight path with 180-deg turns at either end. A research team member walked beside the participants to ensure their safety and stood by them when they turned.

### 2.6 Experimental Protocol 3: RSFC

Participants were instructed to remain as motionless as possible in a seating position for 6 min in a dimly lighted space. Participants were asked to close their eyes, remain relaxed, and refrain from concentrating on anything specific. A research team member monitored participant conduct during the trial to ensure they did not doze off. In addition, after the trial, participants were asked if they had fallen asleep at any moment. None of the participants reported that they had fallen asleep.

### 2.7 fNIRS Data Processing and Analysis

#### 2.7.1 Fine motor task

To estimate the HRF elicited by the left and right finger-tapping tasks, the following procedure was implemented.

Data were processed and analyzed in MATLAB R2017b (The MathWorks, Natick, Massachusetts, United States) using Homer3^30^ and custom-made scripts, as illustrated in Fig. 2(b). First, the raw time series went through a quality control process in which readings with very high or meager optical density (OD) changes, low signal-to-noise ratio (SNR), or very long source-detector separation were excluded from further analysis, using the hmrR_PruneChannels function (ranging from 1e−7 to 1e7, SNRthresh = 2, SDrange = 0.45). Then, the raw data were transformed into units of change in OD (ΔOD). Motion artifacts were identified on a channel-by-channel basis using the hmrR_MotionArtifactByChannel function. Briefly, if the signal amplitude was larger than 0.5 (AMPthresh) or the standard deviation augmented by a factor of 15 (SDthresh) over a time window of 1 s (tMotion), then a segment of the time series of 1s length (tMask) was identified as motion artifact. The data in the motion-contaminated segments were interpolated by a cubic spline using the hmrR_MotionCorrectSpline function with a smoothing parameter of p=0.99 to avoid corrupting the clean segments adjacent to motion artifacts,^31^^,^^32^ and motion artifacts were corrected using the hmrR_MotionCorrectWavelet with an interquartile range of 1.5.^33^ To ensure the exclusion of any trials with residual motion artifacts, we conducted motion artifact rejection within a −2 to 10 s window subsequent to our motion-artifact correction efforts, specifically to eliminate trials that continued to exhibit motion-related characteristics using the hmrR_StimRejection function. Finally, the OD data were bandpass filtered between 0.01 and 3 Hz to minimize the effect of slow drifts^34^ The ΔOD time-series were converted to oxy-(HbO) and deoxygenated (HbR) hemoglobin employing the modified Beer–Lambert law, implemented in the hmrR_OD2Conc function with a partial pathlength factor of 1 for each wavelength, thus yielding units of μM-mm.^35^ The individual HRFs were estimated via a general linear model (GLM; hmrR_GLM). The analyzed range was from 2 s before stimulus onset to 25 s post-stimulation. The GLM was solved by ordinary least squares.^36^ A consecutive series of 1-s broad Gaussian functions interspaced 1 s was used as the basis function. Channels spaced by less than 1.5 cm were considered short separation, and the short channel with the most significant correlation was used to regress the superficial hemodynamics from the estimated HRF.^37^^,^^38^ A polynomial correction of order three was implemented.

#### 2.7.2 Two-min walk

Changes in oxygenated hemoglobin (HbO) from baseline standing to walking were used as a proxy for cortical activation.^39^ The same procedure described in the fine motor task paradigm was implemented to process the fNIRS signals. Although individual trials in experiments might present limitations, the application of the GLM approach in fNIRS single trial analysis has demonstrated robustness, even in one-shot experiments.^40^

#### 2.7.3 Resting-state

After motion artifact detection and correction, as described in the paragraphs above, the remaining clean segments were then bandpass filtered into the FC band (0.009 to 0.08 Hz) using the hmrR_BandPassFilt function^41^^,^^42^ and converted to hemoglobin concentrations.

### 2.8 Functional Connectivity

FC analysis was performed only on the time points identified as free of motion artifacts; discarding the motion-artifact segments has been shown to preserve interhemispheric FC.^32^ A procedure to remove spontaneous fNIRS fluctuations common to all channels was implemented using short separation channel (SSC) signal regression. First, the SSC signal s was computed as the average time course of all short channels $s_{i}(t)$ (n=2 in our case): $s(t)=\frac{1}{n}\overset{n}{\underset{i}{\sum }}y_{i}(t)$. Then, the SSC signal was regressed from the measured fNIRS time-series Y(t) using a Tikhonov regularized estimator:

$$\beta_{g}=[s(t{)}^{T}s(t)+\lambda^{2}I{]}^{-1}s(t{)}^{T}Y(t),$$ (1)

where the regularization parameter was chosen as $\lambda =0.001\;\max\{\mathrm{diag}[s(t{)}^{T}s(t)]\}$.^41^ The residual time-series $Y^{\prime }(t)$ was then computed as follows:

$$Y^{\prime }(t)=Y(t)-s(t)\beta_{g}.$$ (2)

$Y^{\prime }(t)$ generates channel-based connectivity matrices, computing the Pearson correlation r between all channels. To obtain an index of intrahemispheric connectivity, channel-to-channel correlation coefficients in the left and right hemispheres, respectively, were averaged separately for each participant.^43^ An index of interhemispheric connectivity was calculated by averaging all channels and their contralateral homologs.

### 2.9 Statistical Analysis

A paired sample t-test was used to compare the mean change in hemoglobin during the pre-task baseline period and the task period to assess channel activation.^44^ Comparisons between PD patients and controls were made through a non-parametric Wilcoxon–Mann–Whitney test for the task-based paradigms because part of the data was not normally distributed, according to a Shapiro–Wilk test.^45^ Five different metrics extracted from the hemodynamic response were compared between controls and PD patients. Furthermore, relationships between fNIRS data from channels demonstrating statistically significant group differences and clinical variables (Table 1) were examined with Spearman’s correlation coefficients, and corrections for false positives were carried out using a false-discovery rate (FDR) adjustment.^46^^,^^47^ To further investigate if the side of onset mediates the correlations between clinical variables and fNIRS features, all data from PD patients were stratified according to the side of onset (11 left and 9 right), and then, a partial correlation analysis was conducted to assess the relationship between HRF features and clinical variables while controlling for age and years of education.

**Table 1:** Demographic and clinical features of PD patients and controls. Unless otherwise indicated, the mean values are given, with standard deviations in parenthesis.

	PD group	Control group	p-Value
Age (years)	69.9 (10.1)	65.6 (9.9)	0.23
Proportion of men (%)	60	45	0.52
Education (years)	9.3 (4.1)	12.1 (5.2)	0.06
Reported handedness (right/left)	20/0	20/0	1.00
MoCA	21.6 (3.8)	26.2 (2.7)	<0.001
Unified PD rating scale III	37.4 (18.6)	N/A	N/A
Disease duration (years)	8.2 (3.8)	N/A	N/A
Freezing of gait questionnaire (range)	2.7 (0 to 15)	N/A	N/A
Levodopa equivalent daily dosage (mg)	707.5 (394.8)	N/A	N/A
HY scale (range)	2.9 (2 to 4)	N/A	N/A
Side of symptom onset (right/left)	9/11	N/A	N/A

For the intra and interhemispheric FC, Pearson’s coefficients were transformed to a Fisher z-score using $Z(r)=\frac{1}{2}\;\ln[\frac{1+r}{1-r}]$ before computing the Wilcoxon–Mann–Whitney tests.^48^

In all experimental paradigms, corrections for multiple comparisons within-subject and between-group were performed using the FDR adjustment.^46^^,^^47^ Categorical variables were compared using the Chi-square test.

## 3 Results and Discussion

### 3.1 Clinical Evaluation

Table 1 summarizes the clinical and demographic characteristics of the studied groups. No significant differences were found in age, proportion of male participants, years of education, or body mass index. However, the cognitive dysfunction of PD patients was significantly higher than that of control participants (p=0.0002), as shown by previous studies.^49^^–^^51^ Because cognitive impairment is common in patients with PD and can occur at any stage of the disease,^52^ those with mild cognitive impairment were included in this study.

### 3.2 Cortical Activation during Fine Motor Movement

**Fig. 3:**
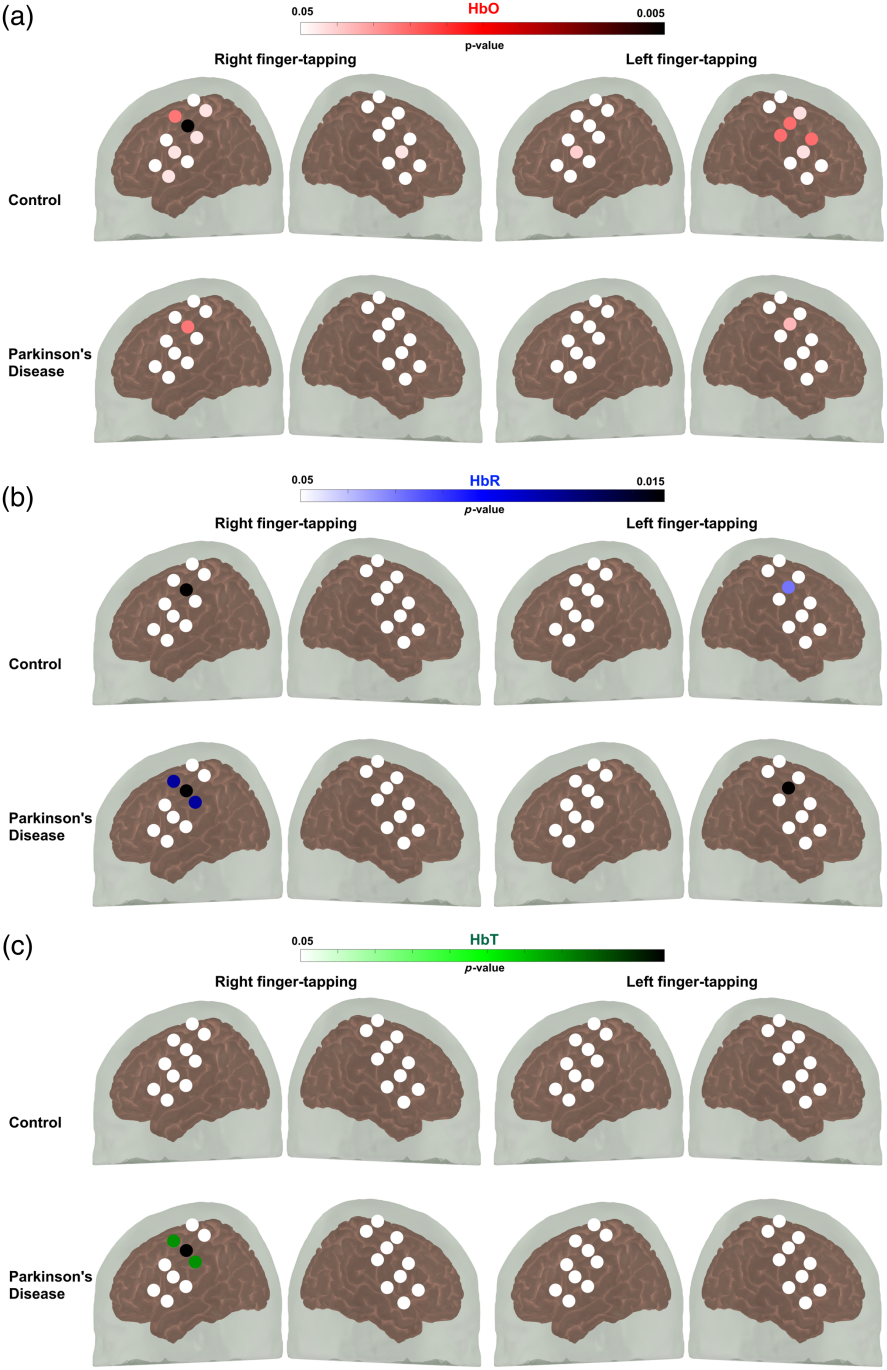
Paired sample t-test was used to compare the mean change in hemoglobin during the pre-task baseline period and the task period to assess channel activation. The color gradient indicates the p-value during the finger-tapping task. Channels in white demonstrate no statistically significant variations in hemoglobin levels between the pre-task baseline and task periods. (a) Oxyhemoglobin (HbO), (b) deoxyhemoglobin (HbR), and (c) total hemoglobin (HbT).

Channels that showed significant hemodynamic activation in the motor cortex evoked by finger tapping of both the dominant (right) and non-dominant hand (left) are shown in Fig. 3.

Five different features of the HRF were assessed for all hemoglobin types: peak value, time to peak (also known as peak latency), area under the HRF curve (AUC), mean value, and slope from stimulus onset to peak because studies have revealed that the peak latency and form, which vary across and within persons for each hemoglobin signal, task, and brain area, may be significant variables to research.^53^^,^^54^ For example, a larger time-to-peak in the HRF means it takes longer for the HbO concentration to peak following neuronal activation.

**Fig. 4:**
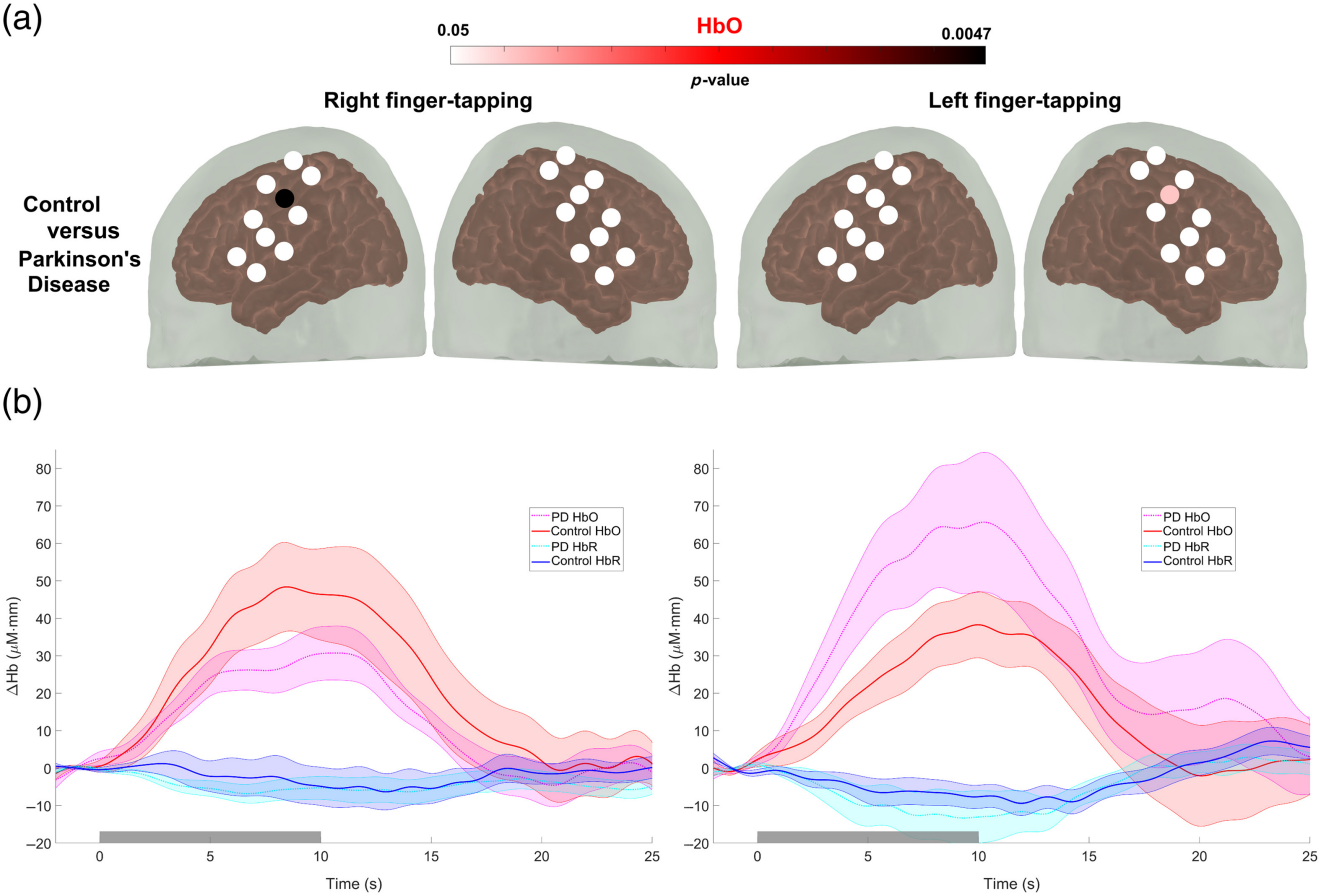
Group comparisons. (a) A Wilcoxon–Mann–Whitney test with the FDR correction for false positives was used to detect group differences in mean oxyhemoglobin (HbO) changes during the finger-tapping task. The color gradient indicates a p-value of group differences. White channels indicate no statistically significant differences between the two groups. No significant differences were found in either HbR or HbT. (b) Average waveforms at channel 15 (right finger tapping) and channel 10 (left finger tapping). The gray rectangle denotes stimulus onset and duration.

Channel-by-channel comparisons revealed significant peak latency differences between the PD and control groups in channel 15 for right finger tapping and channel 10 for left finger tapping, as shown in Fig. 4(a) (see Table S1 in the Supplementary Material for correlation values corrected for false positives). The results depicted in Fig. 4(b) indicate that patients with PD had delayed hypoactivation in the motor cortex during the fine motor task with the dominant hand and delayed hyperactivation with the non-dominant hand, as measured by near-infrared spectroscopy. This delayed activation may explain the difficulty of patients with PD to perform bimanual movements, as revealed by Wu et al.^55^ using fMRI. Furthermore, our findings are supported by previous studies that have found hypoactivation in the contralateral primary motor cortex elicited with a simple finger-tapping task in patients with a similar HY stage.^56^

### 3.3 Correlation Analysis during Fine Motor Movement

**Fig. 5:**
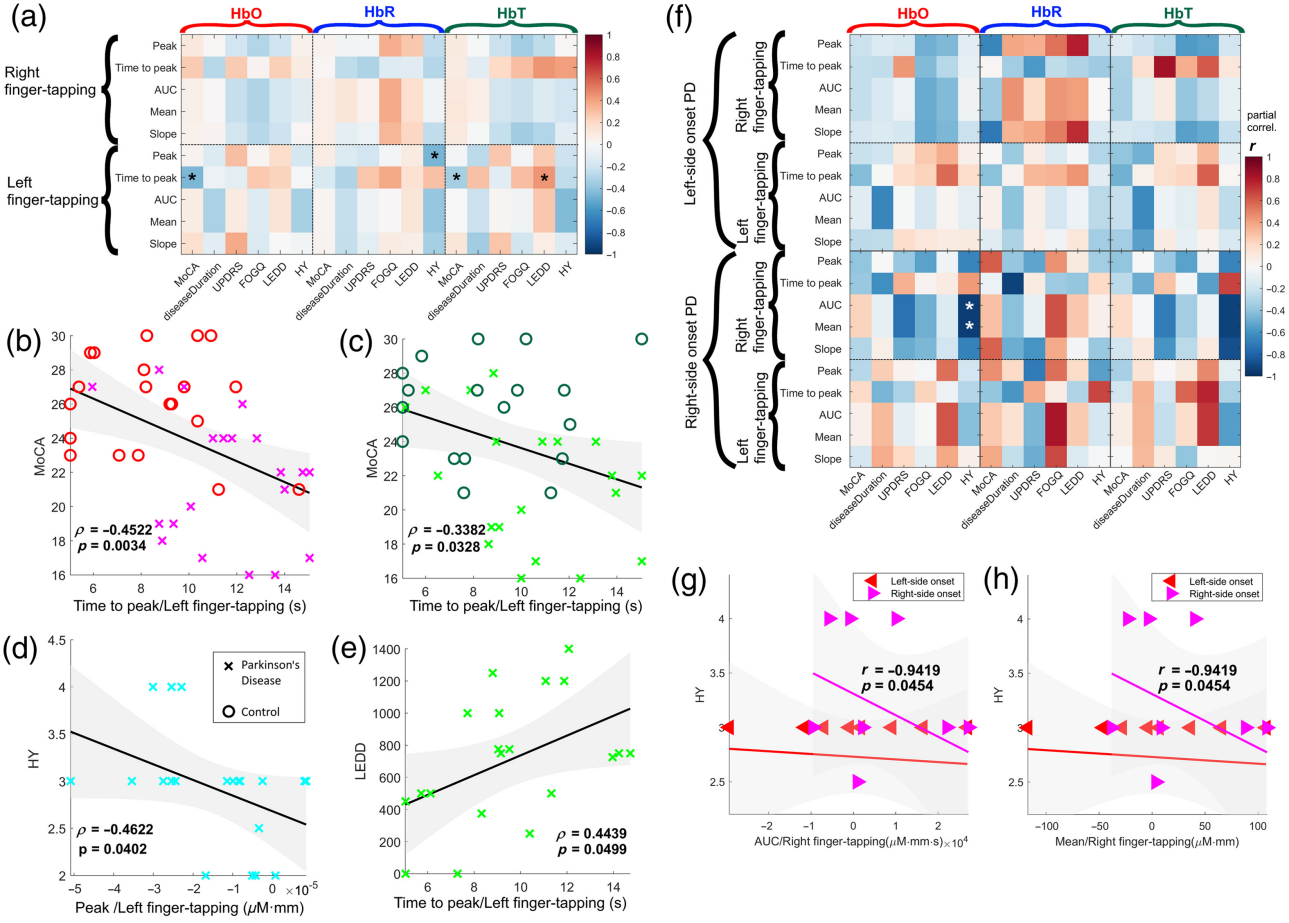
Correlation analysis. (a) Correlation matrix between clinical variables and fNIRS-derived metrics. The asterisk sign (*) denotes a significant relationship (p<0.05). (b) Correlation between MoCA score and time-to-peak during left finger tapping (HbO). (c) Correlation between MoCA scores and time-to-peak during right finger tapping (HbO). (d) Correlation between HY scale and peak amplitude of left finger tapping (HbR). (e) Correlation between l-dopa equivalent daily dose and time-to-peak during left finger tapping and (f) correlation between MoCA scores and time-to-peak during left finger tapping. Shaded areas denote 95% confidence intervals.

Our findings suggest significant correlations among various measures of hemodynamic activity in the motor cortex using fNIRS and different cognitive and clinical variables. After considering only channels with a significant difference between groups and correcting correlations for false positives, only the activity on channel 10, corresponding to left finger tapping, showed statistically significant correlations with clinical variables. A breakdown of each of the correlations is shown in Fig. 5 (see Table 2 for correlation values corrected for false positives).

**Table 2:** Correlation values between HRF features and clinical variables. Significant values after the FDR correction are displayed in bold.

	Spearman’s correlation	HbO						HbR						HbT					
		MoCA	disease duration	UPDRS-3	FOGQ	LEDD	HY	MoCA	disease duration	UPDRS-3	FOGQ	LEDD	HY	MoCA	disease duration	UPDRS-3	FOGQ	LEDD	HY
Right	Peak	0.11	0.01	-0.19	-0.30	-0.20	0.05	0.04	-0.03	-0.02	0.36	0.27	-0.25	0.09	-0.01	-0.22	-0.34	-0.26	0.02
	Time to Peak	0.26	-0.28	0.26	0.03	0.24	0.09	0.01	-0.25	-0.31	-0.13	-0.12	-0.29	0.08	-0.11	0.21	0.30	0.39	0.41
	AUC	0.06	0.01	-0.24	-0.31	-0.18	-0.12	0.07	0.12	0.04	0.37	0.17	-0.04	0.11	0.07	-0.19	-0.22	-0.14	-0.20
	Mean	0.06	0.01	-0.24	-0.31	-0.18	-0.12	0.07	0.12	0.04	0.37	0.17	-0.04	0.11	0.07	-0.19	-0.22	-0.14	-0.20
	Slope	0.05	0.03	-0.19	-0.28	-0.21	0.01	0.07	-0.09	-0.06	0.33	0.21	-0.25	0.10	-0.01	0.24	-0.35	-0.30	-0.11
Left	Peak	0.03	-0.14	0.30	0.02	0.18	-0.18	0.08	-0.14	-0.20	-0.03	0.08	**-0.46**	-0.02	-0.26	0.27	-0.03	0.26	-0.21
	Time to Peak	**-0.45**	-0.07	-0.10	0.26	0.22	-0.02	-0.16	-0.30	0.28	0.38	0.14	0.30	**-0.34**	0.29	-0.11	0.30	**0.44**	-0.25
	AUC	0.07	-0.32	0.09	-0.13	0.14	-0.32	0.02	-0.23	-0.04	0.01	0.12	-0.38	0.00	-0.22	0.02	-0.04	0.27	-0.43
	Mean	0.07	-0.32	0.09	-0.13	0.14	-0.32	0.02	-0.23	-0.04	0.01	0.12	-0.38	0.00	-0.22	0.02	-0.04	0.27	-0.43
	Slope	0.17	-0.11	-0.11	-0.15	0.00	-0.03	0.03	-0.24	-0.10	0.09	0.06	-0.33	0.12	-0.35	0.28	-0.18	0.07	-0.13

The correlation between time to peak during left finger tapping of both HbO and HbT and MoCA is moderate (r=−0.4522, r=−0.3382) and significant (p=0.0034, p=0.0328, respectively); this stronger negative relationship between the time it takes for HbT and HbO to peak during a motor task with the non-dominant hand and cognitive performance possibly indicates that cognitive processes require optimal oxygen and blood supply to the brain^57^ and impairments of cerebral oxygenation present in PD patients have an effect on cognitive performance.

The association between peak HRF during left finger tapping of HbR and HY scale is moderate (r=−0.4622) and significant (p=0.0402), indicating a negative relationship between the peak of HbR in the right hemisphere and the severity of PD symptoms. Previous works supported this finding; for instance, Junqué et al.^58^ pointed out that asymmetries have been reported in PD, with left-hemisphere functions relatively more preserved than right-hemisphere ones; this suggests that the right hemisphere may play an important role in PD symptoms. In addition, although not explicitly addressing PD, Goldstein et al.^59^ suggested that normal aging has a functionally asymmetric effect on the right hemisphere, which could potentially contribute to PD symptoms. However, further research is needed to confirm this potential negative relationship between the amplitude of deoxyhemoglobin in the right hemisphere and the severity of PD symptoms.

The correlation between time to peak during left finger tapping of HbT and levodopa equivalent daily dose is significantly moderate (r=0.4439, p=0.0499), suggesting a significant positive relationship between the time it takes for total hemoglobin to peak in the right hemisphere and medicament dosage. This may be explained by the effect of levodopa absorption and delivery to the brain.^60^ No association was seen in disease duration, UPDRS score, or FOGQ.

The correlation between AUC during right finger tapping and the HY scale was found to be statistically significant: r(17)=−0.9419, p=0.0454, indicating a strong negative association between the two variables, as shown in Figs. 5(f)–5(g). The correlation coefficient of −0.9419 suggests that, after accounting for age and education, an increase in the HY scale is associated with a decrease in the AUC of the HRF in patients with right-side onset but not in patients with left-side onset. The same results were obtained with the mean value of the HRF during right finger tapping and the HY scale [Fig. 5(h)]. This supports a neural inefficiency hypothesis in which patients with more disability show a decrease in the activity evoked by finger tapping with the dominant hand. This result may be explained by the limited sample size and the fact that all patients with an HY of 4 are patients with right-side onset. Our results contrast with the findings of Zhu et al.,^61^ in which left-dominant symptom patients showed more severe gait impairments as well as more severe sleep-related dysfunction.

### 3.4 Motor Cortex Activation during Walking

**Fig. 6:**
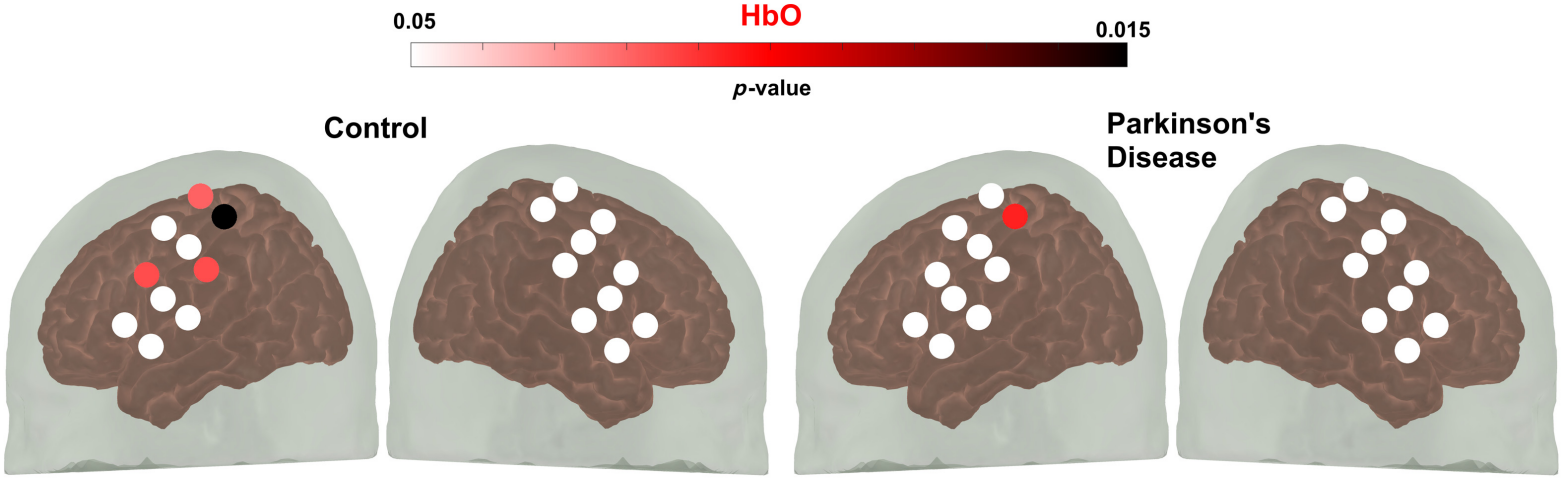
Paired sample t-test was used to compare the mean change in hemoglobin during the pre-task baseline period and the walking task period to assess channel activation. The color gradient indicates the p-value during the walking task. Channels in white demonstrate no statistically significant variations in hemoglobin levels between the pre-task baseline and task periods.

There was a significant increase in activity in the primary motor cortex for the simple walking task compared with baseline in both the control group (four channels, t(19)=2.93, p<0.01) and the PD group (one channel, t(19)=2.46, p<0.03), as depicted in Fig. 6. All active channels were found in the left hemisphere. However, there were no significant differences between patients with PD and controls; this could be explained due to two findings. First, normal walking does not significantly increase blood oxygenation in the PFC,^62^ and although it is expected in the motor cortex, it was not observed in our data. We hypothesize this may be due to technical limitations of the fNIRS probe used, such as suboptimal optode separation. Second, many studies reported that increased cortical activity during walking has confounding effects due to motion artifacts.^63^ Further research is required to determine its causes.

### 3.5 FC Analysis during Walking

**Fig. 7:**
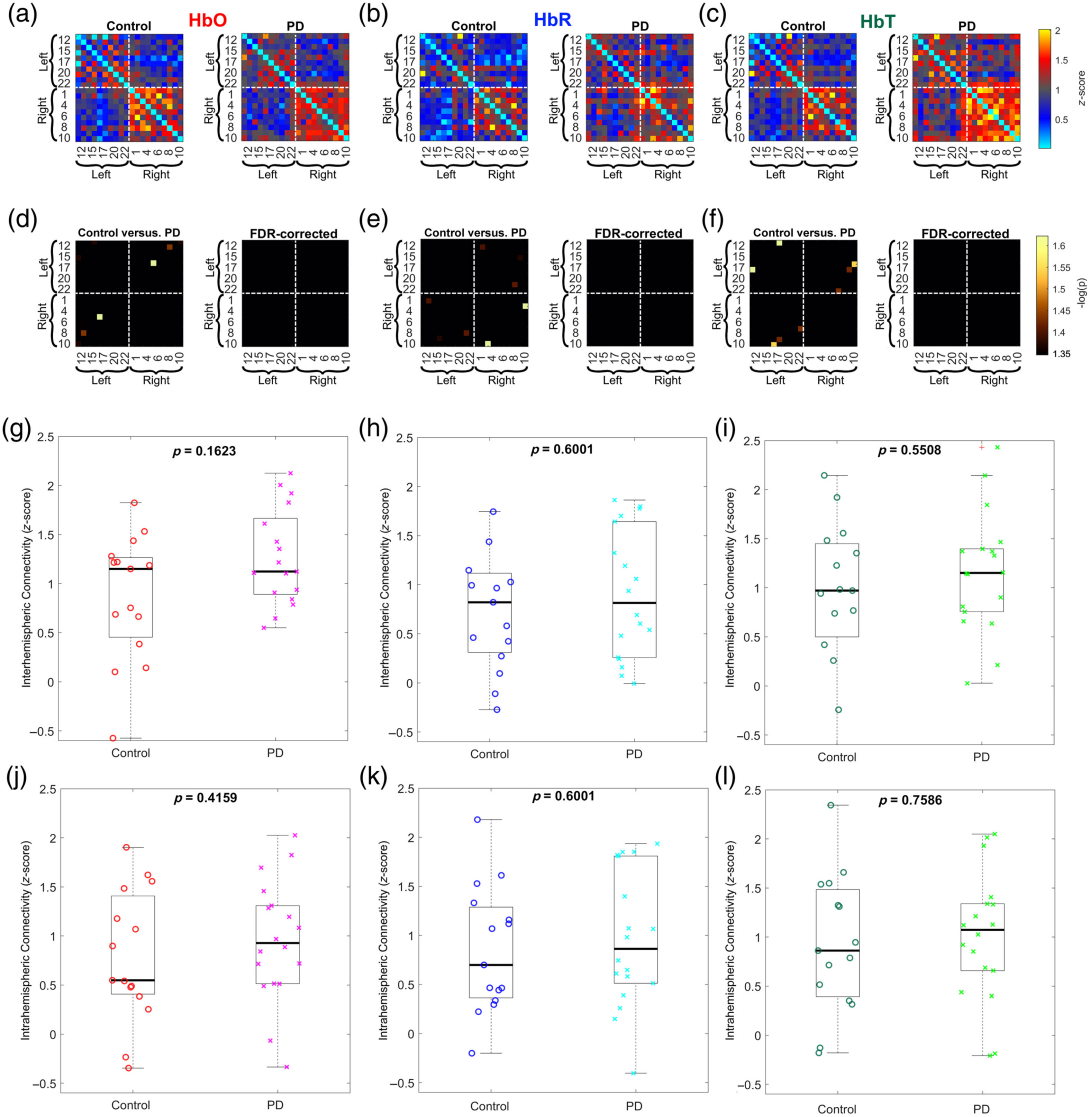
FC during walking: (a)–(c) color-coded average FC matrices (Fisher’s z-score) between channels for both groups calculated for HbO, HbR, and HbT, respectively. Warm colors denote positive correlations and cold colors denote negative correlations. (d)–(f) Statistical comparison between controls and PD patients. Only statistically significant correlations are shown (p<0.05). After the FDR correction, no significant differences were found. (g)–(i) Group comparison of interhemispheric connectivity computed for HbO, HbR, and HbT, respectively. (j)–(l) Group comparison of intrahemispheric connectivity computed for HbO, HbR, and HbT, respectively.

Several factors might explain the lack of significant differences in FC observed in the study comparing patients with PD and the control group during a walking task (Fig. 7). The inherent variability in task execution and the heterogeneity of the condition could result in increased variability in observed FC patterns, potentially masking any subtle differences.

### 3.6 RSFC Analysis

**Fig. 8:**
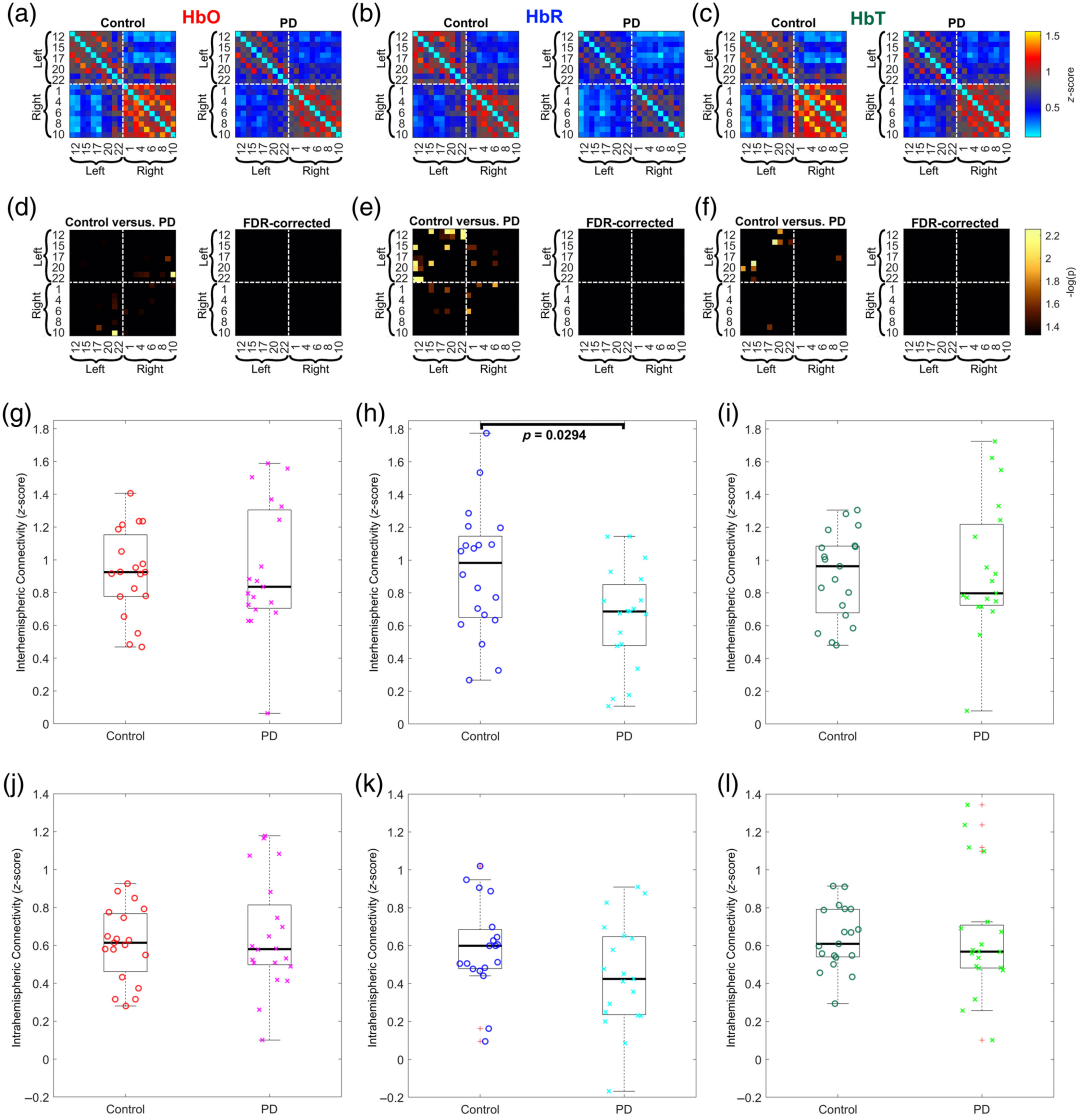
FC: (a)–(c) Color-coded average FC matrices (Fisher’s z-score) between channels for both groups calculated for HbO, HbR, and HbT, respectively. Warm colors denote positive correlations, and cold colors denote negative correlations. (d)–(f) Statistical comparison between controls and PD patients. Only statistically significant correlations are shown (p<0.05). After the FDR correction, no significant differences were found. (g)–(i) Group comparison of interhemispheric connectivity computed for HbO, HbR, and HbT, respectively. A statistically significant decrease in interhemispheric connectivity in PD patients compared with controls was found in HbR measurements. (j)–(l) Group comparison of intrahemispheric connectivity computed for HbO, HbR, and HbT, respectively.

We hypothesized that there might be differences in RSFC between PD patients and control participants. The z-score transformed correlation matrices are shown in Figs. 8(a)–8(c). Our analysis revealed significant differences in several channels, particularly in the HbR contrast, as depicted in Figs. 8(d)–8(f). In addition, a general trend of decreased positive connections in the PD group was observed. However, these results did not survive the FDR correction. This trend of decreased connectivity in PD patients confirms observations made in previous studies.^64^

Our analysis revealed significant differences between the two groups regarding the strength of interhemispheric connectivity, as shown in Figs. 8(g)–8(i). More specifically, a statistically significant decrease in interhemispheric connectivity in PD patients compared with controls was found in HbR measurements [see Fig. 8(h)]. The existing literature studying brain networks has associated a decreased interhemispheric FC with symptoms of PD. Gan et al.^65^^,^^66^ found that PD patients with impulse control disorders had abnormal interhemispheric RSFC, and those patients with levodopa-induced dyskinesia had altered interhemispheric connectivity. The study found that PD patients with freezing of gait had decreased interhemispheric connectivity in certain brain regions compared with PD patients without freezing of gait and healthy controls.^67^ These findings imply the possibility of using decreased interhemispheric FC as one of many potential neuroimaging biomarkers for diagnosing PD, albeit it is shared by other neurodegenerative conditions such as Alzheimer’s disease.^68^

Our intrahemispheric connectivity analysis revealed trends between the groups, but these results did not pass the FDR threshold. Specifically, control participants showed stronger positive connections within a hemisphere than PD patients (see Figs. 8(j)–8(l)].

## 4 Conclusion

During fine motor movement, patients with PD had delayed hypoactivation in the motor cortex with the dominant hand and delayed hyperactivation with the non-dominant hand. This delayed activation may explain the difficulty that patients with PD have performing fine motor movements and may be interpreted in line with the neural inefficiency hypothesis, as the delayed and abnormal activation patterns indicate inefficient neural processing during motor tasks.

A significantly higher cognitive dysfunction in PD patients could further complicate these findings. The cognitive dysfunction noted in PD patients compared with control participants could exacerbate the inefficiencies in neural processing, impacting both motor and cognitive functions.

The significant correlations between measures of hemodynamic activity in the right motor cortex during left finger tapping and cognitive or clinical variables underline the intertwined nature of motor and cognitive impairments in PD, suggesting that any compensatory mechanisms may be limited by concurrent cognitive decline.

Although there were significantly active channels in the left motor cortex for the walking task in both the control and PD groups, there were no significant differences between patients with PD and controls. Our findings suggest that interhemispheric FC is crucial for motor function and that motor impairments are reflected as disruptions in this connectivity. Therefore, both activation in the primary motor cortex elicited by a fine motor task and interhemispheric FC may be proposed as neuroimaging biomarkers for assessing PD. However, caution should be exercised in its interpretation because decreased interhemispheric connectivity is also present in other neurodegenerative diseases, such as Alzheimer’s disease.

The study faced several limitations that may impact the generalizability and depth of the findings. First, the technical constraints of the fNIRS probe, such as suboptimal optode separation, could limit the accuracy of cortical activation measurements, particularly during complex tasks such as walking. Second, the omission of turn registration in the walking task might have overlooked significant aspects of motor function in PD. Finally, the relatively limited sample size encourages further research with enhanced methodologies and larger cohorts to validate these findings comprehensively.

Further research is needed to confirm these results and explore its potential in studying PD.

## Supplementary Material



## Data Availability

The data supporting this study’s findings are available in Zenodo at https://zenodo.org/record/7966830, Ref. 69.
