# Supplementary material for: Parkinson’s disease patients show delayed hemodynamic changes in primary motor cortex in fine motor tasks and decreased resting-state interhemispheric functional connectivity: a functional near-infrared spectroscopy study
Source: Neurophotonics. 2024 May 29;11(2):025004. doi: 10.1117/1.NPh.11.2.025004 (PMC11135928; doi:10.1117/1.NPh.11.2.025004)
Supplement: Supplementary file 1 [file NPh_011_025004_SD001.pdf]

Functional Near-Infrared Spectroscopy Reveals Delayed Hemodynamic Changes in the Primary Motor Cortex During Fine Motor Tasks and Decreased Interhemispheric Connectivity in Parkinson's Disease Patients

Edgar Guevara,[a,b,*] Francisco Javier Rivas-Ruvalcaba,[c] Eleazar Samuel Kolosovas-Machuca,[d] Miguel G. Ramírez-Elías,[d] Ramón Díaz de Leon Zapata,[e] Jose Luis Ramirez-GarciaLuna,[b,f] Ildefonso Rodríguez-Leyva[c]

[a]CONAHCYT-Universidad Autónoma de San Luis Potosí, Mexico
[b] Coordinación para la Innovación y Aplicación de la Ciencia y la Tecnología, Universidad Autónoma de San Luis Potosí, Mexico
[c]Neurology Service, Hospital Central "Dr. Ignacio Morones Prieto", Faculty of Medicine, Universidad Autónoma de San Luis Potosí, Mexico
[d]Faculty of Science, Universidad Autónoma de San Luis Potosí, Mexico
[e]Tecnológico Nacional de México, Campus San Luis Potosí, Mexico
[f]Division of Surgery, Hospital Central "Dr. Ignacio Morones Prieto", Faculty of Medicine, Universidad Autónoma de San Luis Potosí, Mexico

Supplementary Material

Table S1. P-values after false discovery rate correction (FDR) of the group-level difference for two different conditions: right and left finger-tapping. Five different metrics extracted from the hemodynamic response were compared between controls and Parkinson’s disease patients. Significant values after FDR correction are displayed in **bold**.

Supplementary Material

Table S1. P-values after false discovery rate correction (FDR) of the group-level difference for two different conditions: right and left finger-tapping. Five different metrics extracted from the hemodynamic response were compared between controls and Parkinson's disease patients. Significant values after FDR correction are displayed in **bold**.

		Peak			Time to peak			AUC			Mean			Slope		
Finger-tapping	Channel	HbO	HbR	HbT	HbO	HbR	HbT	HbO	HbR	HbT	HbO	HbR	HbT	HbO	HbR	HbT
Right	13	0.7500	0.9705	0.9892	0.2686	0.8735	0.4402	0.9191	0.7623	0.9246	0.9447	0.7623	0.9246	0.9676	0.8187	0.8627
	12	0.7500	0.9705	0.8541	0.5843	0.3905	0.4402	0.9461	0.7710	0.9246	0.9461	0.7710	0.9246	0.3207	0.8187	0.2548
	17	0.7500	0.9705	0.4971	0.2686	0.9891	0.8272	0.9191	0.7710	0.7587	0.9447	0.7710	0.7587	0.3207	0.8811	0.2548
	16	0.9461	0.9705	0.8627	0.2686	0.8735	0.4402	0.9461	0.9892	0.9246	0.9461	0.9892	0.9246	0.4076	0.8811	0.4434
	**15**	0.9461	1.0000	0.7081	**0.0047**	0.8735	0.4402	0.9191	0.7710	0.9246	0.9447	0.7710	0.9246	0.3207	0.9676	0.3010
	22	0.9461	0.9705	0.8627	0.5705	0.3905	0.7471	0.9191	0.7710	0.6832	0.9447	0.7710	0.6832	0.4076	0.8187	0.6344
	21	0.7500	0.9705	0.4971	0.8604	0.3048	0.6645	0.9191	0.5678	0.6628	0.9447	0.5678	0.6628	0.3889	0.8187	0.2548
	20	0.7500	0.9705	0.6738	0.8604	0.3905	0.5002	0.9191	0.8858	0.9246	0.9447	0.8627	0.9246	0.9676	0.9676	0.8817
	19	0.7500	0.9705	0.4545	0.8604	0.8494	0.8392	0.9191	0.5678	0.4679	0.9447	0.5678	0.4679	0.5117	0.8187	0.2839
	18	0.7500	1.0000	0.4545	0.8604	0.8735	0.6645	0.9191	0.7710	0.6628	0.9447	0.7710	0.6628	0.4076	0.9676	0.3010
Left	1	0.8423	0.9560	0.9927	0.0505	0.5251	0.2546	0.5376	0.9892	0.7473	0.5376	0.9892	0.7473	0.8858	0.9892	0.9246
	2	0.9892	0.9560	1.0000	0.8497	0.5251	0.7556	0.9676	0.7473	0.7557	0.9461	0.7473	0.7557	0.7754	0.6948	0.9246
	4	0.9892	0.7205	0.9927	0.3593	0.5251	0.6638	0.6470	0.7473	0.7473	0.6470	0.7473	0.7473	0.6470	0.6948	0.9246
	5	0.8423	0.9560	0.9927	0.5278	0.5251	0.7556	0.6257	0.7473	0.7473	0.6257	0.7473	0.7473	0.6470	0.9892	0.9246
	6	0.9892	0.9560	0.9927	0.5524	0.5251	0.7556	0.9676	0.7473	0.7473	0.9461	0.7473	0.7473	0.9031	0.9892	0.9246
	7	0.9892	0.9560	0.9927	0.7944	0.2136	0.6638	0.9676	0.7473	0.7473	0.9461	0.7473	0.7473	0.8858	0.9892	0.9246
	8	0.9892	0.9892	1.0000	0.3593	0.5251	0.2546	0.9676	0.9892	0.7557	0.9461	0.9892	0.7557	0.6470	0.8750	0.9246
	9	0.6011	0.9560	0.9927	0.4164	0.5251	0.6638	0.5376	0.7473	0.7473	0.5376	0.7473	0.7473	0.6470	0.6948	0.9246
	**10**	0.9892	0.9560	1.0000	**0.0450**	0.9352	0.2546	0.6570	0.7473	0.7473	0.6570	0.7473	0.7473	0.6470	0.8750	0.9246
	11	0.8423	0.9560	0.9927	0.7944	0.5251	0.6638	0.5731	0.7473	0.7473	0.5731	0.7473	0.7473	0.7754	0.7133	0.9246
